# Toxins of Locus of Enterocyte Effacement-Negative Shiga Toxin-Producing *Escherichia coli*

**DOI:** 10.3390/toxins10060241

**Published:** 2018-06-14

**Authors:** Maike Krause, Holger Barth, Herbert Schmidt

**Affiliations:** 1 Department of Food Microbiology and Hygiene, Institute of Food Science and Biotechnology, Garbenstrasse 28, University of Hohenheim, 70599 Stuttgart, Germany; maike.krause@uni-hohenheim.de; 2 Institute of Pharmacology and Toxicology, University of Ulm Medical Center, Albert-Einstein-Allee 11, 89081 Ulm, Germany; holger.barth@uni-ulm.de

**Keywords:** LEE-negative STEC, Shiga toxin, Subtilase Cytotoxin, Cytolethal distending toxin, EHEC-Hly, profiling studies, O104:H4, O113:H21, TS18/08

## Abstract

Studies on Shiga toxin-producing *Escherichia coli* (STEC) typically examine and classify the virulence gene profiles based on genomic analyses. Among the screened strains, a subgroup of STEC which lacks the locus of enterocyte effacement (LEE) has frequently been identified. This raises the question about the level of pathogenicity of such strains. This review focuses on the advantages and disadvantages of the standard screening procedures in virulence profiling and summarizes the current knowledge concerning the function and regulation of toxins encoded by LEE-negative STEC. Although LEE-negative STEC usually come across as food isolates, which rarely cause infections in humans, some serotypes have been implicated in human diseases. In particular, the LEE-negative *E. coli* O104:H4 German outbreak strain from 2011 and the Australian O113:H21 strain isolated from a HUS patient attracted attention. Moreover, the LEE-negative STEC O113:H21 strain TS18/08 that was isolated from minced meat is remarkable in that it not only encodes multiple toxins, but in fact expresses three different toxins simultaneously. Their characterization contributes to understanding the virulence of the LEE-negative STEC.

## 1. Introduction

*Escherichia coli* strains that produce Shiga toxins (Stx) occur widely in the gastrointestinal tract of animals and humans. In most animals, the presence of Shiga toxin-producing *E. coli* (STEC) does not cause any disease (reviewed in [1]). In humans however, a STEC subgroup, the enterohemorrhagic *E. coli* (EHEC) can cause serious, life-threatening diseases such as hemorrhagic colitis or the hemolytic uremic syndrome (HUS) [2]. Clinically relevant EHEC strains produce one or more Stx which are considered to be crucial for the development of HUS [3]. Many EHEC strains also express additional virulence factors required to cause disease. The majority of these are encoded by phages, plasmids, and pathogenicity islands (PAIs) (reviewed in [4,5,6]). Amongst them, the locus of enterocyte effacement (LEE) is frequently detected in EHEC strains [7,8,9]. This 35.6 kb large pathogenicity island contains genes encoding a type III secretion system and several translocated effector proteins, including the translocated intimin receptor (*tir*), as well as the adhering intimin (encoded by *eae*) [10,11,12,13,14,15]. The LEE is responsible for the formation of attaching and effacing (AE) lesions in intestinal epithelial cells. They are caused by changes in the cytoskeleton and the accumulation of actin at the attachment site [16,17]. The interactions between bacterial and epithelial cells are mediated by the bacterial outer membrane protein intimin and the binding of its receptor, Tir, which is translocated into the epithelial cell via the type III secretion system [15]. Furthermore, the LEE is a mobile genetic element that can be transmitted by horizontal gene transfer [18]. Due to its causality for sequelae and its rate of detection in STEC, samples that are analyzed for STEC properties and found to encode Stx are by default screened for the *eae* gene, as it is a marker for the occurrence of the LEE [19,20]. Particular EHEC serogroups such as O157, O26, O103, O145, and O111 are associated with human infections and as such are sometimes called the big five serogroups [21]. Following the recommendation of the European Food Safety Authority (EFSA), beside these five serogroups, EHEC belonging to the serogroup O104 are considered to be potentially highly virulent and together, these six are the most important serogroups that should be screened for in stool samples from patients [22].

Besides these typical EHEC, a growing number of LEE-negative EHEC have been isolated from stool samples. This showed on the one hand that the LEE is not mandatory for infections and on the other hand raised the question whether they are as pathogenic as the LEE-positive ones [9,23,24]. In addition, numerous STEC isolates from animals, food, and the environment were shown to be LEE-negative [25,26,27]. For instance, of all isolates originating from infections in Switzerland during the periods between 2000 to 2009 and 2010 to 2014, about 30% did not contain the LEE [7,9]. This leads to the question: why do these strains not contain the LEE? Until today, several studies have shown that the LEE-negative STEC are not a clonal group but evolved independently by acquiring comparable virulence features in parallel [27,28]. In their study on the location of the insertion sites of LEE, Bertin et al. [19] identified three different tRNA genes—*selC*, *pheU*, and *pheV*—which were used for chromosomal insertion. They suggested that these sites could additionally be used for the insertion of other foreign DNA. Based on a comparative study of mainly LEE-negative food isolates and 12 strains of the HUSEC strain collection, Hauser et al. [29] showed in a study in 1996, that in the majority of the food isolates—96% of the characterized strains—the three integration sites for LEE were occupied by different DNA insertions, which could be the cause of the missing LEE [23]. Still, the analyzed strains clustered together with pathogenic strains based on multi-locus sequence typing (MLST), which allowed for them to be classified as a potential cause of infections in humans [29]. Current theories state that these STEC harbor alternative factors such as the *saa* (STEC autoagglutinating adhesin), *sab* (for STEC autotransporter (AT) mediating biofilm formation), *iha* (Irg A homologue adhesion), and/or *tia* (toxigenic invasion loci A) gene, probably to cope for the functional loss of LEE and convey pathogenicity on a different path [30,31].

Two of these alternative adhesins—Saa and Sab—were identified within an LEE-negative EHEC of the serotype O113:H13, which caused a HUS-associated outbreak in Australia [32,33,34]. Saa is a plasmid-encoded adhesin which showed minor similarities with the adhesin YadA from *Yersinia enterocolitica* (about 25% amino acid identity) and the phage-encoded immunoglobulin binding protein EibD of *E. coli* [32,35,36]. Initial analyses indicated that the functionality depends on the length of C-terminal 3′ repeats of the Saa protein [32]. So far, however, these findings could not be verified in follow-up studies. The capacity of Saa to allow *E. coli* laboratory strains to adhere to HEp-2 cells (Human Epithelial type **2** cells) was not significantly dependent on the length of the 3′ repeats and/or the expression level of Saa [37,38].

The *sab* gene encodes a large protein of about 160 kD that resembles features of the AT protein family. This family covers the entirety of surface proteins which are either linked to the outer membrane or released by proteolysis of Gram-negative bacteria. [33]. Each AT protein reveals a characteristic layout of three different domains. Beginning at the N-terminal signal peptide domain, the proteins compose a passenger domain of various functions (α-domain), and end with a conserved pore-forming domain at the C-terminus [33,39]. Sab is located on the surface and was found to contribute to the adhesion to HEp-2 cells and biofilm formation on polystyrene surfaces of the pathogen *E. coli* O113:H21 and laboratory strain *E. coli* JM109 by Herold et al. [33].

Tarr et al. [40] described the *iha* gene which mediates adherence in an *E. coli* O157:H7 strain and non-adherent *E. coli* laboratory strains. It encodes a protein of 78 kDa which is homologous to the adhesive protein IrgA (iron regulated gene A) of *Vibrio cholerae* [40,41,42]. In contrast to *saa* and *sab*, *iha* can be found in LEE-negative and LEE-positive STEC as well as other pathogenic *E. coli* [38,43]*.* Based on these genetic profiling studies, it is assumed that *iha* is an adhesion-associated gene which seems to be preserved throughout STEC serotypes. This indicates a probable importance for human infections [38,40,43]. However, deletion of neither *saa* nor *iha* showed a non-adhering phenotype, which is why it is assumed that there are multiple factors involved in the adherence of LEE-negative STEC [32,40].

The *tia* gene was originally described for enterotoxigenic *E. coli* (ETEC) in the form of an invasion factor for several intestinal cell lines [30,31]. Nevertheless, *tia* can also be found in LEE-negative STEC, where it is encoded within a pathogenic island termed SE-PAI [44]. In their in vivo study involving two different LEE-negative STEC, Bondì et al. [45] showed that *tia* is needed to mediate the invasion into Caco-2 (human colon adenocarcinoma cells/Cancer coli-2) and HEp-2 cells. The transformation of a *tia*-carrying expression vector to an *E. coli* laboratory strain did not lead to a comparable effect on cell invasion, though. This indicates that the presence of Tia is not sufficient to transfer the invasive property of an STEC strain [45].

Including all HUS cases, there were 1885 EHEC infections registered in Germany in 2016 [46]. Since only the detection of Stx or the *stx* gene is crucial for a therapeutic decision, isolations of STEC from stool samples and subsequent enrichment cultures were only infrequently performed [47]. As a consequence, there were only 348 STEC isolates serotyped in 2016 [46]. Within these more thoroughly characterized strains, the majority belonged either to serogroup O157 (48 isolates) or O91 (47 isolates). This is of great interest for two main reasons. First, O157 strains are typically LEE-positive and cause severe infection processes in humans [48]. Second, O91 strains predominantly belong to the group of LEE-negative STEC and are typically classified as less infectious/dangerous [22,49], although an O91 strain that caused HUS and was shown to produce a variant of Stx2 has been described [50]. In addition, O91 strains have been regularly isolated from patient samples and could be found in different foods [7,27,49,51,52]. Recent studies showed that O91 isolates of various origin (food, environment, and humans) share similar genotypic profiles, are phylogenetically related, could be mainly attributed to cattle as a reservoir for human infections, and, thus, could potentially lead to severe human infections [53,54]. Comparing the infection numbers and distribution in detected serotypes of 2016 to the three previous years, there is an increase in the number of cases, but the overall distribution of LEE-positive and LEE-negative pathogens remained steady throughout those years [46,55,56,57]. With respect to the large STEC outbreak in Germany in 2011 which was caused by an *E. coli* O104:H4 strain that carried *stx*_2a_ and lacked the *eae* gene, these data show that the impact of LEE-negative STEC on human health has to be taken seriously. In this context, it is noteworthy that this particular strain originates from enteroaggregative *E. coli*, which most likely evolved to an STEC by acquiring a bacteriophage that encodes *stx*_2a_ [58,59,60]. Finally, there is a demand to understand the complexity of the virulence factor profiles of this subgroup of STEC.

The goal of this review is to enlighten the variety of the pathogenicity factors of LEE-negative STEC targeting especially actively secreted proteins that have a toxic effect on host cells. Based on examples of recent studies on the virulence profiles in LEE-negative STEC, the general procedure and results of such studies are presented. In the following, the functionality and regulation of the toxins that are found in LEE-negative STEC are described.

## 2. Toxins of LEE-Negative STEC

More than 80 studies on the genotyping und virulence profiling of STEC and EHEC were published in the past 10 years, examples of which are given in several studies [23,61,62,63]. In general, all of these screening studies aimed to elucidate the serotypes and virulence properties of STEC including Stx-variants, LEE, further toxins, and other adhesion factors such as the ones mentioned above. As the sources of EHEC infections are manifold, the origin of the examined strains varied from environmental and animal isolates to food isolates to clinical isolates [64,65,66]. Among them, cattle are one of the main reservoirs for STEC. Hence, isolates originating from bovine meat and dairy products, as well as the production plants of bovine products and workers in this field were often examined [26,65,67,68,69,70,71,72]. Furthermore, animal products originating from sheep, goat, deer, or wild boar are of particular interest for screening STEC virulence profiles because these animals harbor a large amount of potential EHEC strains [73,74,75,76,77,78]. In addition to the investigation of animal products, fresh products such as lettuce and spinach are also typically examined in such screening studies [51,79].

Each study followed an essentially similar track of identification. When starting from a sample pool, the first step was to detect *stx* genes and particularly the *eae* gene by polymerase chain reaction (PCR). A *stx-*positive PCR was then usually followed by an isolation of the strain that harbored the *stx* gene [23,70,80]. Subsequently, the serotypes and genetic profiles regarding further toxins and pathogenicity factors were examined. Among these studies, subtyping of the *stx* variants and the detection of enterohemolysin (EHEC-Hly) were the most common targets of investigation [81,82,83]. Besides these, the presence of other toxin genes, such as the cytholethal distending toxin V (Cdt-V) and the subtilase cytotoxin (SubAB), was often determined [62,67,84,85,86]. Furthermore, the identification of adhesion factors, which are present in addition to or alternatively to the LEE, were of great interest. Here, especially *iha* and *saa*, and less often *sab*, were targets for screening [26,87,88].

Slanec et al. [23] isolated 34 STEC strains out of 504 food samples to identify their specific virulence profiles. The *stx* subtype (*stx*_1_ and *stx*_2_, and their variants) and the virulence genes *eae*, *ehxA*, *espP*, as well as the type III effectors *nleA* and *cif*, the adhesins *iha* and *efa1*, and the toxin genes *subAB* and *cdt-V* were investigated. The authors showed that the majority of the strains (88%) carried *stx*_2_ variants. Among these, 18% harbored a second toxin gene, while 4% carried even two additional toxin genes that were different from *stx*. Two *stx* variants, independent of the combination of *stx*_1_ with *stx*_2_, or two different *stx*_2_ alleles were found in 29% of all isolates. Of those, 38% carried *subAB* in addition to two *stx* genes. Ignoring the differentiation into *stx*_1_ and *stx*_2_ subtypes, this study identified as many as 33 different virulence types [23]. In contrast to the results of Slanec et al. [23], Martins et al. [89] identified 45 virulence profiles when ignoring the *stx* type in 65 isolates originating from sheep. Here, 56% carried just one *stx* gene, 33% two *stx* genes, and 11% three. Furthermore, the toxin genes *subAB* and *cdt-V* were analyzed. These genes were carried by 2% and 3% of the analyzed strains, respectively. In addition to the aforementioned genes, the author also investigated *eae*, *ehxA*, *saa*, and *iha*. The studies by Slanec et al. [23] and Martins et al. [89] had already conclusively demonstrated the complexity and intricacy of virulence gene profiling in STEC. In addition, the distribution of percentages of the quantity of *stx* genes and all other tested genes reveal that even the statistical distribution of virulence genes is highly variable in different strain sets.

The study by Slanec et al. [23] highlights a major drawback of all virulence screening studies. Typically, all screenings are conducted on a genetic level and do not determine if the encoded toxins and virulence genes were actually expressed. Only a few studies performed biochemical and microbiological tests to show the presence of the toxin that was identified on a genetic level. Often, Vero cell assays were performed to characterize the phenotypic features of isolates and show the presence of Stx in the corresponding culture supernatant [89]. As an alternative method, quantitative real-time polymerase chain reactions (qRT-PCR) could also be conducted to identify expression levels of different *stx* genes within one strain. The limitation lies in the differentiation between two variants of one Stx class [90]. The expression of enterohemolysin is typically proved with hemolysis assays on special blood agar plates [86].

**Figure 1:**
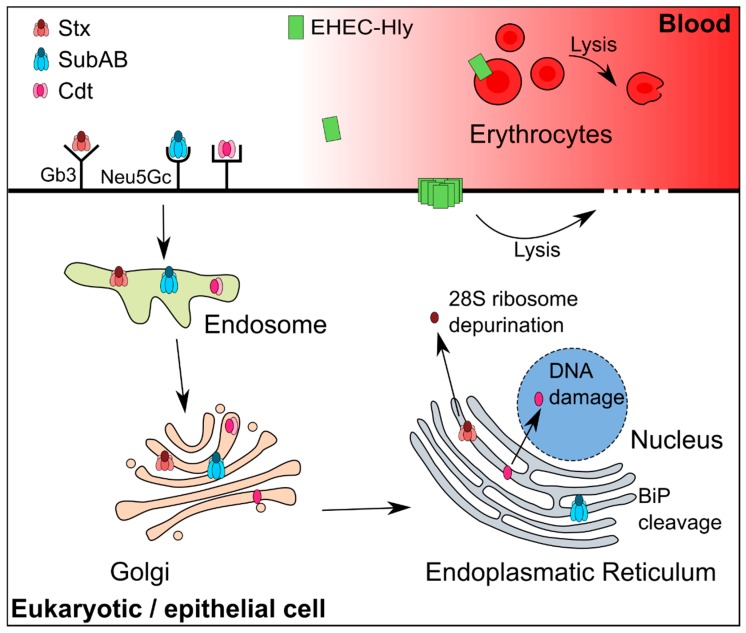
Schematic view of the transport and target sites of the toxins Stx, SubAB, Cdt, and EHEC-Hly. Gb3 and Neu5Gc stand for globotriaosylceramide and N-glycolylneuraminic acid and are the binding partners of Stx and SubAB on the cell surface, respectively. The cell membrane receptor for Cdt is not known and thus not indicated here. SubAB cleaves its natural substrate, the endoplasmatic chaperone BiP (binding immunoglobulin protein) in the endoplasmatic reticulum. Stx depurinates the 28S subunit of the ribosome in the cytosol. Cdt leads to DNA damage in the nucleus and EHEC-Hly causes lysis of erythrocytes and epithelial cells. The effect of EHEC-Hly on target cells mediated by the OMV associated form is not depicted. Modified according to Beddoe et al. [95] and Jinadasa et al. [96].

Nevertheless, all screening studies revealed a great variation in virulence profiles which often encode multiple toxins. This leads to the question how these multiple toxin systems could work. Following the assumption that the information located in the bacterial chromosome is essential, it is likely that each of the toxins encoded is expressed. To our knowledge, there are almost no data or studies available that target the question of multi-toxin expression and their regulation. Still, there has been data obtained on the regulation of some of the toxins found within the repository of LEE-negative STEC. In the following, the currently known characteristics, functions, and regulations of the virulence factors Stx, SubAB, EHEC-Hly, and Cdt are summarized to allow for a glimpse into the complexity of a regulation system that has to manage multiple toxins. Table 1 gives an overview of the toxins that are described in detail in the following sections and highlights their function, strain of origin, and alternative species in which they could be found. A schematic overview of the transport and target sites of Stx, SubAB, Cdt, and EHEC-Hly are presented in Figure 1. These toxins are frequently found in LEE-negative STEC and sparked great interest to understand their potential virulence [23,85,87,88,89].

### 2.1. Stx

As main pathogenicity factors of EHEC, the Stx family is currently one of the best described toxin families. In 1983, O’Brien et al. [91] identified a verotoxin, produced by an *E. coli* O157:H7 strain, to be similar to the previously reported Stx that is encoded by *Shigella dysenteriae* type 1. Following this recognition, the molecular characterization of Stx in pathogenic *E. coli* revealed that there are two main groups of Stx, namely Stx1 and Stx2. Based on genetic variations and the resulting differences in toxicity, toxin receptors, and amino acid compositions, the two main variants were further subtyped into Stx1a to Stx1d, and Stx2a to Stx2f [61,97,98,99,100]. In both cases, the a-form is considered to be the prototype for the respective main variant. Among the variants, Stx1a shows the highest cytotoxicity in Vero cell cytotoxicity assays [101]. Nevertheless, the severe and life-threatening HUS is frequently associated with the production of Stx2a and Stx2c [102].

Stx belong to the class of AB_5_ toxins. They are composed of one enzymatic active A-subunit and a pentamer of B-subunits, which mediates the uptake of the holotoxin via endosomes into target cells [103,104,105,106]. The cytotoxic effect of Stx is based on the irreversible removal of an adenine of the 28S ribosomal subunit, which prohibits the interaction of the elongation factor 1 [107,108,109]. This inhibits the protein synthesis and finally leads to apoptosis of the affected cell [109].

The expression of the members of the Stx family is regulated by many different environmental effects, such as temperature, growth phase, oxidative stress, quorum sensing, and antibiotics [110,111,112,113,114,115,116]. A common regulatory mechanism for Stx expression is the inclusion in regulatory processes of the phage replication cycle, because Stx1 and Stx2 are encoded in the genome of lambdoid prophages.

Its specific location between the genes encoding the late anti-terminator Q and the lysis enzymes allows Stx to be co-transcribed with the late phage genes [117,118]. Basically, this happens when the prophages are induced. Phage induction is connected with the bacterial SOS response, which mediates one of the main regulation pathways for the Stx production. Several antibiotics and hydrogen peroxide also activate the SOS response, thereby likewise increasing Stx production [116,119]. This connection is one of the main reasons why the treatment of an EHEC infection with antibiotics is not recommended, because the increased Stx production can lead to a more severe infection process [120]. In contrast, a negative effect on the Stx expression mediated by the SOS response is induced by nitric oxide. This negative response is caused by the downregulation of RecA, the global initiator of the SOS response [121,122]. In addition, the expression of Stx1 is sensitive to the concentration of iron due to the fact that the *stx*_1_*AB* promotor is iron dependent. Specifically, decreased levels of iron lead to an increase in Stx1 production and vice versa [123].

Although only limited effectors are known for the regulation of Stx1, there are several affecting conditions that were analyzed for the regulation of Stx2. For instance, the global regulator protein H-NS (histone-like nucleoid structuring protein) affects the expression of Stx2 by changing the activity of the specific promoter and the modulation of the phage induction. This effect was shown to depend on temperature, but remain unaltered upon changes in pH, osmolarity, oxygen tension, iron levels, or carbon sources [111,124]. The latest reported global regulator that is involved in the modulation of Stx2 production is the RNA chaperone Hfq. Kendall et al. [125] showed that the production of Stx2 is increased in *hfq* deletion mutants, which leads to the assumption that Hfq itself or small RNAs (sRNA) regulated by Hfq reduce the Stx2 amount in wild-type strains. Furthermore, Stx2 expression appears to be influenced by stress signals and inter-kingdom signaling [126]. Finally, EHEC are able to react to the mammalian stress response by recognizing adrenaline and noradrenaline, which also leads to an increase of its pathogenicity factors including Stx [127].

### 2.2. SubAB

The subtilase cytotoxin (SubAB) was initially described in STEC by Paton et al. [92] in 2001 in the context of a HUS outbreak with *E. coli* O113:H21 98 NK2 in Australia. SubAB is an AB_5_ toxin and has since then been commonly detected in *eae*-negative STEC. Similar to other AB_5_ toxins, SubAB is composed of two subunits. The enzymatically active A-subunit has a subtilase-like serine protease activity, while the pentamer of B-subunits mediates the transport into the target cells in an clathrin-dependent way [128,129]. However, SubAB shows a narrow target spectrum. It only cleaves the endoplasmatic heat shock protein 70 (Hsp70), also named binding immonoglubulin protein (BiP), or 78 kDa glucose-regulated protein (GRP78). The cleavage occurs between its two functional domains, the nucleotide-binding domain (NBD) and the substrate-binding domain (SBD) [130].

Internalization occurs after the B-subunit is bound to its eukaryotic receptor *N*-glycolyl neuraminic acid (Neu5Gc), a glycan side chain that is not synthesized by human cells. Rather, it can be incorporated in the glycan matrix when dairy products and red meat are consumed [95]. Furthermore, experiments by Funk et al. [131] implied a translocation mechanism of the A-subunit (SubA) on its own which is independent of the B-subunit, since in Vero cell-based assays, SubA exhibited cytotoxic effects when applied to the cells in the absence of the B-subunit.

So far, two different encoding locations in the genome of STEC are known for SubAB. SubAB1 was the first identified variant and is located on the virulence plasmid pO113 [92]. During the last eight years, further variants of SubAB were described which are all encoded on the bacterial chromosome, thus named SubAB2-1 to SubAB2-3 [132,133,134].

Despite analyses of several aspects of the biochemical processes involved in the functionality of SubAB and its consequences on the target cells, aspects of the regulation of SubAB expression itself are still unvetted [135,136]. One hypothesis is that the expression is triggered based on the position in the genome. Given that SubAB1 is encoded within a virulence plasmid pO113, it is possible that its regulation is related to the activation of other pathogenicity factors on the same plasmid. Also, the location within the pathogenicity island SE-PAI in close proximity to the *tia* gene of SubAB2-1 or the OEP-locus of SubAB2-2 hints at a regulation that is linked to the genetic surrounding. One exclusive study performed by Hauser et al. [114] gives first insights into the regulation of SubAB1. Based on qRT-PCR data, they reported that the expression of SubAB1 was at its maximum in the late exponential growth phase within an STEC which was isolated from minced meat. This indicates that the expression of the plasmid-encoded SubAB1 depends on the bacterial life cycle [114].

Still, conditions and environmental factors which induce or repress the expression of the SubAB variants are not yet characterized and have to be investigated further to allow for an overall assessment of their pathogenic potentials.

### 2.3. EHEC-Hly

The enterohemolysin or EHEC hemolysin (EHEC-Hly) that has been analyzed in detail is a heat labile toxin which belongs to the repeats-in-toxin (RFX) family [137,138,139]. Therefore, it is characterized by repeats of glycine-rich nonapeptides that are located in proximity to its carboxy-terminus [140,141].

EHEC-Hly is encoded in most EHEC serotypes, which are regularly associated with the development of HUS, the most severe course of an EHEC infection [138]. The actual involvement of the toxin in this infection process was proven by the detection of EHEC-Hly specific antibodies in patients who were suffering from HUS [138,142]. Moreover, it can be found in *eae*-negative STEC, which cause diarrhea in humans, in isolates from livestock such as cattle and sheep, and isolates from foodstuff [23,143,144,145].

After secretion by the type I secretion system, the nonapeptide repeats bind Ca^2+^ ions. In turn, this leads to the activation of the toxin via a conformational switch and the stabilization of the terminal domains [146,147]. Finally, this concludes with the exposure of a hydrophobic domain which is located at the *N*-terminus region and required for the toxic effect itself. It therefore mediates pore formation in the target cells [138]. Whether pore formation occurs in an oligomeric form, such as that described for the *Staphylococcus aureus*’ α-hemolysis, or in a monomeric way is still unknown. The same is true for the mechanism which is needed to form the first contact with the membrane of erythrocytes and eukaryotic cells, both of which are the typical target cells of EHEC-Hly [148,149]. In addition, and as something that is similar to all toxins of the RFX family, EHEC-Hly requires post-transformational activation by fatty acylation of specific lysine residues [150]. Because it is highly conserved throughout the STEC family and is present in most EHEC isolates, it has been proposed as a detection marker for EHEC [151].

EHEC-Hly occurs in two different forms. On the one hand as a free form and on the other hand as an outer membrane vesicle (OMV)-associated form. Both forms are cytotoxic but show differences regarding their mechanism towards different cell types such as microvascular endothelial and intestinal epithelial cells [86,152]. OMV-associated EHEC-Hly does not lyse the target cells directly, while the free version does. By binding to the OMV, EHEC-Hly is shuffled into the target cells, separated from the vesicle by lysozymes, and—after translocation to the mitochondria—it activates caspase-9-mediated apoptosis [152].

Similar to the aforementioned Stx, several regulatory pathways are known for EHEC-Hly. In a study by Li et al. [124], the authors showed that the expression of EHEC-Hly is regulated by the sigma factor RpoS, H-NS, and the sRNA DsrA. Mutations in the RpoS—the mediator of the general stress response—inhibited the expression of EHEC-Hly. For H-NS and DsrA, Li et al. [124] described that the different regulation pathways depended on the temperature. For instance, H-NS inhibit the expression of EHEC-Hly more efficiently when the temperature is higher (37 °C vs. 30 °C). In contrast, the effect of DsrA could either be direct or indirect. Again, this depended on temperature. At a lower temperature (30 °C) H-NS was involved in the DrsA-triggered regulation, while at higher temperatures (37 °C) DsrA interacted directly. In contrast to the effect of H-NS, DsrA increased the expression of EHEC-Hly by inhibiting H-NS or direct transcription activation [124].

### 2.4. Cdt-V

In 1987, Johnson and Lior [153] described another cytotoxin which they named cytolethal distending toxin (Cdt) in accordance with its effect on CHO (Chinese hamster ovary) cells. One of their colleagues, Anderson, reported only a few months later that Cdt is an important virulence factor in an *E. coli* isolate, which originated from a young patient with a severe course of gastroenteritis infection [144]. During the following years, many variants of the Cdt were identified in multiple genera including *E. coli*, *Campylobacter* sp., *Salmonella* Typhi, *Helicobacter* sp., and several others [43,154,155,156].

Cdts are known to form a large subfamily of AB_2_ toxins and spread throughout a variety of Gram-negative bacteria (reviewed in [157]). Amongst this extensive toxin family, there are five different Cdt variants known at the present day which are only found in *E. coli*. Cdt-I and Cdt-II are encoded by enteropathogenic *E. coli* (EPEC) and were not found in STEC [145,158,159], whereas Cdt-V is the variant which is typically encoded by STEC. Besides Cdt-V, Cdt-III can also be found in STEC, but at a much lower rate [159]. Bielaszewska et al. [159] purified Cdt-III and Cdt-V of *eae*-negative patients isolates and verified the expression of these toxins during the infection process by CHO cell culture tests.

Cdt-V—as with all Cdt variants—consists of the three distinct subunits CdtA, CdtB, and CdtC, which assemble in an AB_2_ formation. In Cdt, the enzymatically active A-subunit is composed of the CdtB chain which itself is highly conserved among the different bacteria [160]. The B-subunit is required for the interaction of the holotoxin with the target cell and is assembled by the CdtA and CdtC chain [161,162]. Cdt-V shows DNaseI activity and, as a response to DNA damage, arrests the cell cycle in the G1- or G2-phase depending on the target cells. Hereby, the cell morphology is distended, which can lead to cell death [160,163].

Multiple pathways and interaction partners which are affected by the Cdts are known [160,163,164,165,166,167]. Nevertheless, there are only few studies that target the regulation of the toxins. Oogai et al. [168] published a prediction of regulated genes by sRNAs in the human oral pathogen *Aggregatibacter actinomycetemcomitans* amongst which Cdt was one of those reported. This concept of regulation was previously described for other toxins, e.g., the alpha-toxin of *Staphylococcus aureus* and could be one possibility for the regulation of Cdt in STEC [168,169].

All of the toxins described above are frequently detected within the virulence profiles of STEC isolates from food, environment, livestock, and in EHEC isolates of patients. In addition, they are often found in combination, forming complex multiple toxin networks. The last part of this review will focus on three particular LEE-negative STEC strains whose characterization could count as novel assessments in the understanding of the virulence of this subgroup of STEC.

## 3. The Lee-Negative O113:H21 Strains 98 NK2 and TS18/08, and the *E. coli* O104:H4 Outbreak Strain

LEE-negative STEC strains are frequently detected within human, animal, food, and environmental isolates. However, these strains were rated as less virulent than STEC carrying the LEE. The European Food Safety Authority (EFSA) classifies LEE-negative STEC as less dangerous for human health and states that the serogroups O157, O26, O103, O145, O111, and O104 in combination with the *stx*_2_ and *eae* genes are associated with a higher risk of developing severe infections [22]. However, there are examples which do not fit into this simplified scheme of assessing the pathogenicity of STEC. In 1999, Paton et al. [34] published a study on an *E. coli* O113:H21 which lacked the LEE but was responsible for a HUS outbreak in Australia. Comparative multi-locus sequence typing (MLST) studies on *E. coli* O113:H21 isolates from all over the world revealed that especially strains originating from Australia comprised sequence type ST-820 rather than ST-223, which was predominantly found for strains of serotype O113:H21. These results also pertain to the strain described by Paton et al. [34] and indicate either the association with virulence or regional clustering of this sequence type [88]. Subsequent studies by Paton et al. [92] on the *E. coli* O113:H21 strain 98 NK2 revealed that it also produces a second AB_5_ toxin, the SubAB, which caused cytotoxic effects in cell culture experiments when the Stx was depleted from the supernatant by adding a recombinant *E. coli* strain which specifically binds Stx. This finding was a novelty as such genetic equipment for any other STEC or EHEC had not been described before. Furthermore, a new field of toxin research was opened which led to the identification of several other variants of SubAB within STEC [76,133,134]. Currently, the evolutional origin of SubAB is not known. Paton et al. [92] hypothesized that it arose from genetic rearrangements of harmless genes that become dangerous when encoded and expressed in parallel, and, as shown by many virulence profiling studies, appear to be encoded exclusively in LEE-negative STEC [23,28,44,134]. *E. coli* O113:H21 strain 98 NK2 in particular contains the genes for Stx2, SubAB1, EHEC-Hly, Saa, and Sab [34].

In contrast to the LEE-negative EHEC studied by Paton et al. [34,92] the LEE-negative strain of the 2011 outbreak in Germany does not share the genetic composition attributed to EHEC. As discussed above, this strain is described as an enteroaggregative *E. coli* strain with a *stx*_2a_ prophage, which in addition carries a plasmid encoding an extended-spectrum beta-lactamase [60]. This *E. coli* O104:H4 revealed a unique pathogenicity, 22% of all documented cases developed HUS. This rate is significantly higher than the number of HUS cases of other EHEC strains, which usually lies between 1–5% [46,55,56,57]. Furthermore, the patients who developed HUS were of uncharacteristic age. Typically, children are more prone to develop HUS upon EHEC infection with the highest rate of incidence among the group of children younger than five years. In 2011, however, the median age of HUS patients was 42 (reviewed in [170]).

Besides these two strains, which have been isolated from human feces, there are also LEE-negative STEC which have not caused any infection so far, but are of scientific interest concerning their genetic virulence equipment. One of these strains is the *E. coli* O113:H21 TS18/08. This strain was originally isolated during the study of Slanec et al. [23] from mixed minced meat and characterized as an STEC of serotype O113:H21. Its virulence profile is determined by the presence of the *stx*_2a_ genes as well as the genes for the toxins SubAB1 and Cdt-V. Furthermore, it encodes EHEC-Hly and Iha [23,114]. To our knowledge, this strain/virulence profile is not linked to any infection, but represents an example for the research field of multiple toxin expression in STEC.

The characterization of TS18/08 on the genetic level and the finding that it encodes three different toxins motivated the researchers to investigate whether the transcription of all toxins occur at the same time and whether SubAB contributes to the overall cytotoxicity of the strain itself [23,114]. By using quantitative real-time PCR (qRT-PCR), the transcription levels at different time points within a batch growth were detected. The mRNA levels for *subA*_1_ and *cdt-V* peaked after 3 h of cultivation in the late exponential growth phase. In contrast, *stx*_2_ revealed the highest mRNA-level within the stationary phase after 6 h. The overall highest transcription level of all three toxins was achieved by *cdt-V* at its transcription maximum, most likely caused by the induction of the *cdt-V* encoding prophage within this strain. The second largest amount of transcripts was allocated to *subAB*_1_ and the smallest amount to *stx*_2_. Analysis on the cytotoxicity to Vero cells revealed that SubAB contributes to the toxicity of this strain. This study showed that each toxin of *E. coli* O113:H21 strain TS18/08 was transcribed and that changes on mRNA levels of the three different toxins corresponded to the growth phase of a discontinuous culture [114]. The transcription maxima at different time points showed different expression profiles, which may indicate alternative regulation patterns for each toxin.

Based on the capacity to code for a second AB_5_ toxin, to cause untypical and high pathogenicity, and to encode and express three toxins of different toxin families—imprinted by the strains *E. coli* O113:H21 98 KN2, *E. coli* O104:H4, and *E. coli* O113:H21 TS18/08, respectively—LEE-negative strains should not be underestimated with respect to their risk for virulence.

## 4. Conclusions

This short review showed the complexity and variability of virulence factors of LEE-negative STEC focusing on the toxins. The typically conducted virulence profiling studies only reveal information concerning the genetic state of an STEC/EHEC. Even if the amount of such data is enormous, there is an increased demand for the analyses of these toxins on the protein level.

The outbreak strains from Australia in 1999 and Germany in 2011 exemplify the potentially severe virulence of LEE-negative STEC. As described above, little is known about the regulation of the expression of the different virulence factors, especially toxins, when represented in one single STEC. Concerning this, it is necessary to further explore the expression and regulation mechanisms of different STEC toxins. In this context, whole genome sequencing could be one major technique upon which these studies could be based. Analyses on the genetic location of regulatory proteins, virulence factors in general, and regulatory binding sites within the genome would allow for an elucidation of possible regulatory networks. In addition, it is indispensable to analyze synergistic effects within multiple toxin networks. Knowing specific regulation mechanisms which are linked to respective metabolic and infectious states of the STEC will likely allow for the exploration of their pathogenicity in general and will lead to an improved assessment of their threat to human health.

## Figures and Tables

**Table 1:** Summary of toxins identified within STEC. Besides their biological/biochemical functions, the strain of origin and other organisms, which could encode the toxin too, are depicted.

Toxin	Function	Strain of Origin	Alternative Organisms	Reference
Shiga toxin	Removal of an adenine of the 28S ribosomal subunit	*Shigella dysenteriae* type I	*E. coli* O157:H7 other EHEC/STEC	[91]
Subtilase cytotoxin	Cleavage of GRP78/BiP	*E. coli* O113:H23 98 NK2	other STEC	[92]
Enterohemolysin	Pore formation in target cells	*E. coli* O157:H7 EDL 933	other EHEC/STEC	[93]
Cdt	DNase I activity	*E. coli* O128:NM	*Campylobacter* spp., *Salmonella* Typhi, *Helicobacter* spp.	[94]
